# A risk prediction model for post-stroke depression in Chinese stroke survivors based on clinical and socio-psychological features

**DOI:** 10.18632/oncotarget.16907

**Published:** 2017-04-07

**Authors:** Rui Liu, Yingying Yue, Haitang Jiang, Jian Lu, Aiqin Wu, Deqin Geng, Jun Wang, Jianxin Lu, Shenghua Li, Hua Tang, Xuesong Lu, Kezhong Zhang, Tian Liu, Yonggui Yuan, Qiao Wang

**Affiliations:** ^1^ School of Information Science and Engineering, Southeast University, Nanjing, China; ^2^ Department of Psychosomatics and Psychiatry, Zhongda Hospital, School of Medicine, Southeast University, Nanjing, China; ^3^ Department of Psychosomatics, The Affiliated First Hospital of Suzhou University, Suzhou, China; ^4^ Department of Neurology, Affiliated Hospital of Xuzhou Medical College, Xuzhou, China; ^5^ Department of Neurology, Nanjing First Hospital, Nanjing, China; ^6^ Department of Neurology, Gaochun People's Hospital, Nanjing, China; ^7^ Department of Neurology, Jiangning Nanjing Hospital, Nanjing, China; ^8^ Department of Psychiatry, Huai'an No.3 People's Hospital, Huai'an, China; ^9^ Department of Rehabilitation, Affiliated Zhongda Hospital of Southeast University, Nanjing, China; ^10^ Department of Neurology, The First Affiliated Hospital of Nanjing Medical University, Nanjing, China; ^11^ The Key Laboratory of Biomedical Information Engineering of Ministry of Education, Institute of Biomedical Engineering, School of Life Science and Technology, Xi'an Jiaotong Univerisity, Xi'an, China

**Keywords:** post-stroke depression, socio-psychological factor, risk prediction model, logistic regression, decision tree

## Abstract

**Background:**

Post-stroke depression (PSD) is a frequent complication that worsens rehabilitation outcomes and patient quality of life. This study developed a risk prediction model for PSD based on patient clinical and socio-psychology features for the early detection of high risk PSD patients.

**Results:**

Risk predictors included a history of brain cerebral infarction (odds ratio [OR], 3.84; 95% confidence interval [CI], 2.22-6.70; *P* < 0.0001) and four socio-psychological factors including Eysenck Personality Questionnaire with Neuroticism/Stability (OR, 1.18; 95% CI, 1.12-1.20; *P* < 0.0001), life event scale (OR, 0.99; 95% CI, 0.98-0.99; *P* = 0.0007), 20 items Toronto Alexithymia Scale (OR, 1.06; 95% CI, 1.02-1.10; *P* = 0.002) and Social Support Rating Scale (OR, 0.91; 95% CI, 0.87-0.90; *P* < 0.001) in the logistic model. In addition, 11 rules were generated in the tree model. The areas under the curve of the ROC and the accuracy for the tree model were 0.85 and 0.86, respectively.

**Methods:**

This study recruited 562 stroke patients in China who were assessed for demographic data, medical history, vascular risk factors, functional status post-stroke, and socio-psychological factors. Multivariate backward logistic regression was used to extract risk factors for depression in 1-month after stroke. We converted the logistic model to a visible tree model using the decision tree method. Receiver operating characteristic (ROC) was used to evaluate the performance of the model.

**Conclusion:**

This study provided an effective risk model for PSD and indicated that the socio-psychological factors were important risk factors of PSD.

## INTRODUCTION

Post-stroke depression (PSD) is considered to be one of the most frequent and important post stroke sequela, with a prevalence ranging from 20% to 65% [1, 2]. PSD has a negative effect on the therapy, survival and resumption of social activities of patients, and also has a high medical care burden [1]. Previous observational studies indicated that PSD usually occurs within a few months of stroke onset [3]. The longitudinal study reported that a prevalence of PSD was increased to 25% between 0 to 3 months after stroke, decreased to 16% between 3 and 12 months after stroke, and would be increased again 1 year after stroke [4]. Thus, it is important to identify patients at high risk of PSD, which will facilitate early prevention and adequate treatment.

There is evidence for both biological and psychological mechanisms in the etiology of PSD. Some researchers propose a biological mechanism in which depression is caused by brain damage that disrupts neural circuits involved in mood regulation [5]. Other studies have focused on stroke-related factors and showed that demographic factors (e.g., age, sex) [6], medical and psychiatric history [7], type and severity of stroke, lesion location [8], and degree of disability are associated with PSD [9]. However, others have suggested that depression is caused by a psychological reaction to the social and psychological stressors associated with stroke [8]. Previous studies demonstrated that personality traits are associated with PSD, they found the patients with higher levels of neuroticism have higher risk of PSD, suggested that the impact of personality traits on depressive symptoms is mediated through illness cognitions and coping styles [5, 6, 10]. Recent studies reported social support is an important predictor to improve functional recovery after stroke [11], and the PSD patients have low social support, indicating that social support systems could help buffer patients at risk [9, 12–14]. Moreover, stroke survivors have a high prevalence of alexithymia and anhedonia, PSD symptoms that cause a high burden to family caregivers. Unfortunately, the factors investigated in these above-mentioned studies only explain a small part of the risks of PSD. Moreover, the evidence for the role of psychological factors involved in PSD is unclear.

Previous studies have investigated general PSD risk factors (sex, history of depression, family history of depression and somatic comorbidity), potential disease-related risk factors (cognitive impairment, lesion location, leukoaraiosis on computed tomography (CT), Rankin score and cognitive score) [15], and established risk predictors for depression in the first year after stroke. Furthermore, a relationship between psychological variables and the presence of depressive symptoms lasting 2 months after stroke was reported in a study that tried to combine demographic, stroke-related factors and psychological factors to identify their influence on PSD [5]. Although these studies focused on risk factors, they did not develop an operational tool for clinical evaluation. To date, few studies have developed a risk model for the early detection of PSD. A PSD Prediction Scale (DePreS) was proposed to assess the risk of PSD in the first week after stroke [7]. It mainly focused on the socio-demographic and stroke-related factors, including a medical history of depression or other psychiatric disorders, hypertension, angina pectoris, and dressing in Barthel Index (BI). This model has a good predictive performance; however, a lack of socio-psychology factors and cognitive factors limits the interpretation and use for PSD patients. In addition, Mclntosh showed that implementation of the Evidence Based Depression Screening and Treatment (EBDST) protocol improved early detection and treatment of PSD patients in hospital [16]. Although the study tried to explore the risk factors for PSD, there was a lack of an assessment criterion to identify the risk of PSD.

Therefore, the aim of the present study was to identify risk factors of PSD from demographic factors, medical history, vascular risk factors, functional status, socio-psychological factors, and neurological and cognitive functions. Moreover, the main purpose of this study was to develop a clinical and comprehensive risk model for the clinical recognition and early prevention of PSD.

## RESULTS

This study recruited 562 stroke patients. At one month after stroke, 226 cases fulfilled the criteria of PSD. The cumulative incidence of PSD was 40.2%. The characteristics of patients are summarized in Table 1. The range of age was between 24 and 84 years with an average age of 64.28 ± 10 years. There were 225 females, and 119 females with PSD, 106 females without depression. There was a significant difference in Body Mass Index (BMI) (*P* = 0.006) between the PSD group and Non-PSD group. Regarding analysis of medical history and vascular risk factors, a history of Brian_CI, hypertension, diabetes, and smoking and drinking were significantly different (*P* < 0.001) between the PSD and Non-PSD groups. For the socio-psychological, neurological and cognitive functional factors, EPQ_E, EPQ_P, EPQ_N, SSRS, TAS, NIHSS, BI and MMSE were significantly different (*P* < 0.05) between the PSD group and Non-PSD group.

**Table 1:** The baseline characteristics of PSD and Non-PSD patients

Characteristic	PSD (n=226)	Non-PSD (n=336)	*P* Value
**Sociodemographics**			
Age, mean (SD), y	64.31 (10.72)	64.25 (9.97)	0.95^a^
Female	119 (52.65)	106 (31.55)	<0.001^a^
Education, mean (SD), y	13.15 (7.88)	13.51 (7.64)	0.59^a^
BMI (SD)	23.05 (3.29)	24.04 (4.76)	0.006^a^
**Medical history and vascular risk factors (n, %)**			
Brain cerebral infarction	121 (53.54)	74 (22.02)	<0.001^b^
Brain cerebral hemorrhage	8 (3.54)	5 (1.49)	0.152^b^
Depression	2 (0.88)	0 (0)	0.16^b^
Trauma	4 (1.77)	7 (2.08)	0.53^b^
Disturbance of consciousness	3 (1.33)	1 (0.3)	0.31^b^
Hypertension	111 (49.12)	207 (61.61)	0.004^b^
Coronary	11 (4.87)	20 (5.95)	0.71^b^
Auricular fibrillation	7 (1.77)	14 (4.17)	0.65^b^
Diabetes	45 (19.91)	98 (29.17)	0.01^b^
Hyperlipemia	11 (4.87)	17 (13.99)	0.54^b^
Thyroid dysfunction	2 (0.88)	0 (0)	0.16^b^
Epilepsy	2 (0.88)	1 (0.3)	0.57^b^
Alcohol drinker	52 (23.01)	109 (32.44)	<0.001^b^
Smoker	76 (33.63)	163 (48.51)	<0.001^b^
**Social psychological factors, mean (SD)**			
EPQ_E	8.23 94.54)	9.76 (4.92)	<0.001^c^
EPQ_P	6.43 (3.38)	4.62 (2.79)	<0.001^c^
EPQ_N	1.29 (5.66)	6.98 (5.70)	<0.001^c^
LES	38.00 (28.32)	29.65 (32.95)	0.36^c^
SSRS	37.69 (7.05)	41.62 (6.70)	<0.001^c^
TAS	60.41 (7.71)	52.86 (9.26)	<0.001^c^
NIHSS	4.3 (4.23)	3.87 (3.33)	0.04^c^
mRS	2.10 (1.56)	2.05 (1.32)	0.32^c^
BI	73.01 (27.48)	77.25 (24.27)	0.01^c^
MMSE	23.64 (5.91)	25.11 (4.75)	<0.001^c^

Note: SD, standard deviation; PSD, post stroke depression; BMI, body mass index; EPQ_E, EPQ_P and EPQ_N, Eysenck Personality Questionnaire with Extraversion/Introversion (E), Psychoticism/Socialisation (P), Neuroticism/Stability (N); LES, Life Event Scale; SSRS, Social Support Rating Scale; TAS, 20 items Toronto Alexithymia Scale; NIHSS, National Institutes of Health Stroke Scale; mRS, modified Rankin Scale; BI, Barthel Index; MMSE, Mini-Mental State Examination.

^a^Student t-tests; ^b^Fisher Exact Test; ^c^MANOVA Test.

**Figure 1:**
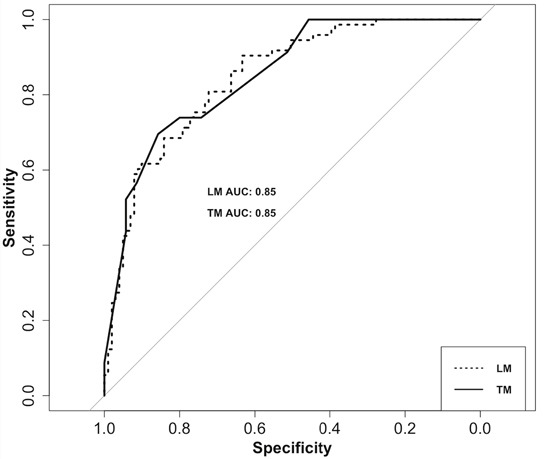
ROC curves of the PSD risk prediction model The dotted line is ROC of logistic model (LM). The AUC of the LM is 0.85 (95% CI, 0.79-0.90), and the accuracy, sensitivity and specificity were 0.76, 0.90 and 0.63, respectively. The solid line is the ROC of tree model (TM). The AUC of the TM is 0.85 (95% CI, 0.75-0.95), and the accuracy, sensitivity and specificity were 0.86, 0.70 and 0.83, respectively.

As shown in Table 2, the multivariable backward regression analysis showed a history of Brain_CI (odds ratio [OR], 3.84; 95% confidence interval [CI], 2.22-6.74; *P* < 0.0001), EPQ_N (OR, 1.18; 95% CI, 1.12-1.25; *P* < 0.0001), LES (OR, 0.99; 95% CI, 0.98-0.99; *P* = 0.0007), SSRS (OR, 0.91; 95% CI, 0.87-0.95; *P* < 0.0001), and TAS (OR, 1.06; 95% CI, 1.02-1.10; *P* = 0.002) were significant predictors for PSD. EPQ_N, LES, SSRS, and TAS were derived from the socio-psychological factors. The model had a good-of-fit with a McFadden value of 0.33. In the Figure 1 (dotted line), the logistic model showed a good discriminatory performance where the area under the curve (AUC) of the receiver operating curve (ROC) was 0.85 (95% CI, 0.79-0.90), and the predictive accuracy, sensitivity and specificity were 0.76, 0.90 and 0.63, respectively.

**Table 2:** Multivariable logistic regression model predicting outcome

Predictors	OR	95% CI	SE	*P* Value
Brain_CI	3.84	2.22-6.74	0.28	<0.0001
EPQ_N	1.18	1.12-1.25	0.03	<0.0001
LES	0.99	0.98-0.99	0.003	0.0007
SSRS	0.93	0.87-0.95	0.02	<0.0001
TAS	1.05	1.02-1.10	0.02	0.002

Note: CI, confidence interval; OR, odds ratio; SE, standard error; Brain_CI, brain cerebral infarction; EPQ_N, Eysenck Personality Questionnaire with Neuroticism/Stability (N); LES, Life Event Scale; SSRS, Social Skills Rating System; TAS, 20 items Toronto Alexithymia Scale.

**Figure 2:**
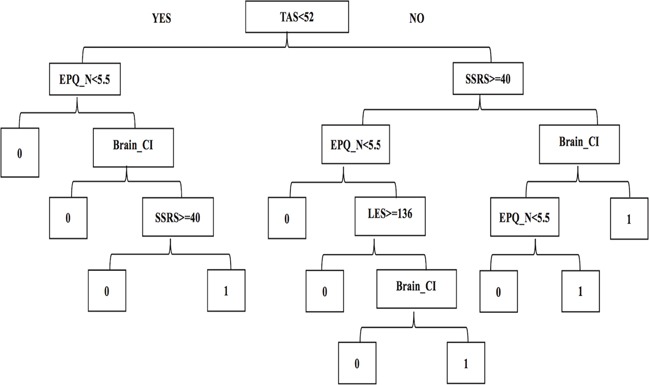
Tree model for the prediction of PSD YES indicates the participant matches the criterion, and to the right indicates the next criterion performed; NO indicates the participant does not match the criterion, and to the left indicates the next criterion performed. Note: 0, the participant has no risk of PSD; 1, the participant has a risk of PSD; Brain_CI, brain cerebral infarction history; EPQ_N, Eysenck Personality Questionnaire with Neuroticism/Stability (N); LES, Life Event Scale; SSRS, Social Support Rating Scale; TAS, 20 items Toronto Alexithymia Scale.

To provide a visualization of the logistic model using the significant predictors, we used the decision tree method to construct a tree model for distinguishing patients with a high PSD risk from the stroke survivors. Figure 2 shows the decision tree with 9 internal nodes and 11 leaves. For each internal node, the split criterion is indicated. The first major split in the tree defines pathways separating high TAS score (>=52) and low TAS score (<52), which is similar to a TAS possible alexithymia cut-off value of 52-60. Within each of these branches, the tree followed the EPQ_N and SSRS assessment at the second level, the EPQ_N and Brain_CI history at the third level, the SSRS, LES and EPQ_N score at the fourth level, and the Brain_CI history at the fifth level. Thus, up to five assessments could identify patients who were at high risk of PSD. The ROC curve in Figure 1 (solid line) of the tree model showed a good performance where the AUC of the ROC was 0.85 (95% CI, 0.75-0.95). The accuracy of the tree model was 0.86.

## DISCUSSION

This study explored potential PSD risk predictors to develop a prediction model, which was used to predict the risk of PSD within 1 month, by the multivariable logistic regression method based on clinical, socio-psychological, neurological and cognitive functional factors. We also generated an evaluation criteria tool for PSD using the decision tree method. The present study yielded two main findings. First, Brian_CI history, EPQ_N, LES, SSRS, and TAS were significant PSD risk factors, of which the last four factors belonged to socio-psychological factors. This suggested that understanding the role of socio-psychosocial factors in the development of PSD is important to improve primary and secondary intervention for stroke survivors. Second, the decision tree provided the critical values of the rule and quantified how they interacted to affect the risk of PSD in stroke survivors.

This study also comprehensively investigated the influence of a broad range of general clinical, socio-psychological, neurological and cognitive functional factors on PSD. Here, we found that only a history of Brain_CI was a significant predictor for PSD by general and clinical data. Previous studies have demonstrated that the correlation between demographic factors and the occurrence of PSD is controversial. Our study did not find demographic factors were risk factors for PSD. However, regarding neurologic deficits, this finding was inconsistent with other clinical studies and meta-analyses that reported that the baseline severity of disability was the most robust correlation with the development of depressive symptoms. A possible explanation was that the patients recruited in our study had mild or moderate nerve dysfunction, and this small difference between the two groups concealed any potential significant influence on PSD. Severe disability caused by large strokes may be an independent predictor of PSD, which is more likely to affect regions involved in mood processing.

For the socio-psychological factors, our findings suggest that certain psychosocial risk factors are important in the development of PSD. The comprehensive life event was estimated with LES, which quantifies the mental stimulation of daily life. This was not entirely in agreement with previous studies of stroke survivors, which reported that comprehensive life event stimulus not a major life event [14, 17], and that negative life event [18] and stressful life event exposure [3, 18] was associated with PSD. Regarding personality traits, stable characteristics were assumed to influence the process of creating the illness cognition. Studies that explored the relationship between personality traits of neuroticism and PSD demonstrated that neuroticism in EPQ might facilitate the onset of PSD and worsen the outcome [10, 19]. The influence of alexithymia, tested by TAS, suggested that stroke survivors had an impaired ability to identify their own negative and positive emotional responses. It also revealed that determining the clinical correlates of emotional unawareness in patients with brain stroke was essential. Furthermore, the predicted role of social support confirmed the lack of social support at admission and was associated with the onset of PSD at 3-months follow-up [9]. Good social support was a protective factor for subsequent PSD and was associated with better post-stroke functioning [20]. In contrast, poor social support including living alone, and absent partners or close friends was identified as a risk factor for PSD [21]. In addition, psychological therapy with good antidepressant effects indirectly demonstrated the important role of socio-psychological factors in PSD [22].

Compared with previous studies [7, 15], the strength of the present study was the combination of various factors, and the use of the decision tree to reveal the relationship between socio-psychological characteristics and the risk of PSD. The tree model generated simple decision rules to assess the risk of PSD to assist the clinical diagnosis. This type of tree model for PSD risk may be particularly useful for rapid assessment. Among the 11 rules, three main and long preventive rules were identified: TAS-EPQ_N-Brain_CI-SSRS, TAS-SSRS-EPQ_N-LES-Brain_CI and TAS-SSRS-Brain_CI-EPQ_N. The study demonstrated a correlation between socio-psychological factors, and revealed their comprehensive involvement for the risk of PSD. Thus, our study used a tree method to demonstrate complex interactions of predictors that might be difficult or impossible to discover using traditional regression techniques. Furthermore, the decision tree showed a better performance for sensitivity (0.70), specificity (0.83), and AUC (0.85), and displayed a reliable distinguishing function for high-risk populations. The decision criteria successfully identified subgroups of patients who require different assessment tests or treatment strategies to achieve optimal medical outcomes. Moreover, compared with the AUC of the logistic model ROC, the predictive performance was similar between the logistic model and tree model. However, the tree model is more simple and intuitive than the logistic model, and the tree model could be convenient application in the clinic. The logistic regression model is used commonly to determine risk factors in medical researches and diagnose [23]. But the logistic model cloud not provide a selection strategy to achieve acute identify. When the logistic model is translated into a tree model, which could be easily converted into convenient If-Then rules [24]. Thus, the tree model could be a symbolic representation and lends itself to easy interpretation by humans.

The present study had some limitations. First, our results cannot be generalized for all stroke features such as biochemical indices and lesion location, which are also considered risk factors [15]. Future studies should combine these to reveal the interactions of pathophysiology risk factors. Second, the predictors used in our models were assessed at 1-month post-stroke, at which point full depressive symptoms may not be present. Further research of PSD at different times may provide clearer information of the risk predictors and improve the effectiveness of the model. Third, the decision rule and exact cut-off point were used to decide individualized optimal diagnostic estimation; however, they are not absolute. Future studies are needed to optimize the model and enhance its accuracy.

In conclusion, we constructed a comprehensive risk prediction model of PSD in Chinese stroke survivors based on their clinical and socio-psychology features. The model indicated that socio-psychological factors are important for identifying the risk of PSD within 1 month and contribute to post-stroke rehabilitation. Furthermore, a decision tool was developed to help clinicians identify the risk of PSD early, which will allow the optimization of PSD prevention strategies in personalized medicine.

## MATERIALS AND METHODS

This study was approved by the Medical Ethics Committee for Clinical Research of ZhongDa Hospital Affiliated to Southeast University. Written consent forms were obtained from the participants or their legal guardians and the study methods were carried out in accordance with the approved guidelines.

### Participants

A total of 562 participants including 226 PSD patients and 336 stroke patients without depression were recruited between May 2013 and Dec 2014, at 8 cooperation hospitals (Affiliated ZhongDa Hospital of Southeast University, The Affiliated First Hospital of Suzhou University, Nanjing First Hospital, Affiliated Hospital of Xuzhou Medical College, Gaochun People's Hospital, Jiangning Nanjing Hospital, Huai'an No.3 People's Hospital, and The Affiliated First Hospital of Nanjing Medical University). To qualify, the patients were required to meet the following criteria: (1) participants had ischemic stroke and intracerebral hemorrhage as determined by CT or Magnetic Resonance Imaging (MRI) data; (2) the age of onset was under 80 years; (3) participants were free of other major psychiatric disorders, including schizophrenia, bipolar disorder, substance abuse (caffeine, nicotine and alcohol), neurodegenerative illness, severe physical illnesses and other medical illnesses; (4) participants were free of anosognosia, neglect, hemianopia, cortical blindness, amnesia, aphasia, dementia and other symptoms hindered assessment.

In a prospective study conducted in China on stroke survivors, the prevalence of PSD was 27.4% two weeks after stroke, and a prevalence of 28% of PSD in survivors within one month after the stroke [25, 26]. In the study, diagnostic evaluations of PSD were carefully conducted for all participants who fulfilled the following diagnostic criteria at three time points (1 week, 2 weeks and 1 month after stroke), combined with previous clinical literature, by two trained senior psychiatrists. Five factors were included in the diagnostic criteria: (1) had stroke before, or stroke occurred earlier than depressive symptoms; (2) met at least two other depressive symptoms with core criterion symptoms of depressed mood and loss of interest or pleasure in nine symptoms of major depressive disorder in Diagnostic and Statistical Manual of Mental Disorders, Fourth edition (DSM-IV); (3) impaired fitness for personal or work functioning; (4) depressive symptoms lasting more than one week; and (5) free of other major psychiatric disorders, including schizophrenia, bipolar disorder, and substance abuse (caffeine, nicotine and alcohol).

### Measurements

All participants underwent assessments at base line including: (1) demographic data (name, age, sex, education, BMI); (2) medical history, and vascular risk factors (smoking history, alcohol consumption, brain cerebral infraction (Brain_CI), brain cerebral hemorrhage, depression, trauma, disturbance of consciousness, hypertension, coronary, auricular fibrillation, diabetes, hyperlipemia, thyroid, and epilepsy); (3) functional status post-stroke (measured using the National Institutes of Health Stroke Scale (NIHSS) [27], BI [28], and the modified Rankin Scale (mRS) [29]); (4) socio-psychological factors (20 items Toronto Alexithymia Scale (TAS) [30], Life Event Scale (LES) [31], Eysenck Personality Questionnaire (EPQ) with four dimensions: Extraversion/Introversion (E), Neuroticism/Stability (N), Psychoticism/Socialisation (P) and lie (L) [32] and Social Support Rating Scale (SSRS) [33])); and (5) cognitive function testing with the Mini-Mental State Examination (MMSE) [34]. A total of 28 variables were considered as potential risk factors for PSD (summarized in Table 1).

### Statistical analysis

All analyses were conducted using statistical package R (version 3.2.3 [2015-12-10]) with the psych, MASS, pscl, c50, pROC and boot packages. Comparisons of demographic and clinical characteristics between the PSD and Non-PSD groups were performed with Student t-tests for continuous variables and Fisher exact tests for categorical variables. To compare the socio-psychological factors between PSD and Non-PSD groups, we performed normality test with Lilliefors test method [35] and homogeneity test of variances with Bartlett method [36]. If the factors satisfied the normalization and homogeneity of variances, we used multivariate analysis of variance (MANOVA), and then, we used one-way ANOVA analysis to estimate the significant differences between the PSD and Non-PSD group; otherwise, we used Kruskal-Wallis test method. The statistical significance threshold was set at *P* < 0.05.

We used a 2-step procedure to develop the risk prediction model of PSD. The patients were randomly divided into two subsets: training set (n=393, 70% of total data), and testing set (n=169, 30% of total data) [37]. First, a multivariable backward logistic regression analysis was performed in the training set to explore the baseline variables that independently predicted the occurrence of depressive symptoms at 1-month post-stroke. The predictors with statistical significance (P < 0.05) were introduced into the final logistic model with a backward procedure. To maintain the validity of selection in the previous steps, the ORs with 95% CIs of the factors were maintained in the final model. Furthermore, goodness-of-fit of the model was measured using McFadden's pseudo R^2^ statistic (McFadden), where a range of 0.2-0.4 represented a very good fit [38, 39].

Second, we quantified the predictive performance of the final logistic model. The predictive accuracy of the model was calculated by a 10-fold cross-validation in the testing set. In addition, predictive discrimination was estimated in the testing set using the AUC of the ROC, where a higher AUC indicated better discrimination.

Third, to construct a clinical predictive model and generate predictive criteria, we converted the regression model into a tree model using the decision tree method in the training set. Decision tree analyses quantifies the association of predictors [40, 41], defines the most efficient pathway to obtaining a dichotomous ruling [42], and produces graphical outputs that summarize the interactions in a visual, easily interpretable format [43, 44]. In the study, we used the rpart package in R to build a classification tree model that graphically depicted quantitative relationships between predictors and PSD risk. The tree was pruned to its optimal size, minimizing both classification error and tree complexity. Moreover, the AUC of the ROC and the accuracy, which was calculated in the testing set, were applied to evaluate the performance of the tree model.

Of note, 3.1% of the data were missing. Missing values were substituted through multiple imputations to reduce bias and to increase statistical power. The imputation technique involved creating multiple copies of the data and replacing missing values with imputed values based on a suitable random sample from their predicted distribution. We used the mice package of the statistical package R.
